# Combined Immunodeficiency Due to *MALT1* Mutations, Treated by Hematopoietic Cell Transplantation

**DOI:** 10.1007/s10875-014-0125-1

**Published:** 2015-01-28

**Authors:** Divya Punwani, Haopeng Wang, Alice Y. Chan, Morton J. Cowan, Jacob Mallott, Uma Sunderam, Marianne Mollenauer, Rajgopal Srinivasan, Steven E. Brenner, Arend Mulder, Frans H. J. Claas, Arthur Weiss, Jennifer M. Puck

**Affiliations:** 1 Department of Pediatrics, University of California San Francisco School of Medicine, and UCSF Benioff Children’s Hospital, Box 0519, 513 Parnassus Avenue, HSE-301A, San Francisco, CA 94143-0519 USA; 2 Department of Medicine, Rosalind Russell Medical Research Center for Arthritis and Howard Hughes Medical Institute, University of California San Francisco School of Medicine, San Francisco, CA 94143 USA; 3 Department of Plant and Microbial Biology, University of California, Berkeley, CA 94720-3102 USA; 4 Innovations Labs, Tata Consulting Services, Hyderabad, AP India; 5 Department of Immunohematology and Blood Transfusion, Leiden University Medical Centre, Leiden, The Netherlands

**Keywords:** BCL10, bone marrow transplant/hematopoietic cell transplant, CARD11, CARMA1, combined immunodeficiency (CID), erythroderma, immune dysregulation

## Abstract

**Purpose:**

A male infant developed generalized rash, intestinal inflammation and severe infections including persistent cytomegalovirus. Family history was negative, T cell receptor excision circles were normal, and engraftment of maternal cells was absent. No defects were found in multiple genes associated with severe combined immunodeficiency. A 9/10 HLA matched unrelated hematopoietic cell transplant (HCT) led to mixed chimerism with clinical resolution. We sought an underlying cause for this patient’s immune deficiency and dysregulation.

**Methods:**

Clinical and laboratory features were reviewed. Whole exome sequencing and analysis of genomic DNA from the patient, parents and 2 unaffected siblings was performed, revealing 2 *MALT1* variants. With a host-specific HLA-C antibody, we assessed MALT1 expression and function in the patient’s post-HCT autologous and donor lymphocytes. Wild type *MALT1* cDNA was added to transformed autologous patient B cells to assess functional correction.

**Results:**

The patient had compound heterozygous DNA variants affecting exon 10 of *MALT1* (isoform a, NM_006785.3), a maternally inherited splice acceptor c.1019-2A > G, and a *de novo* deletion of c.1059C leading to a frameshift and premature termination. Autologous lymphocytes failed to express MALT1 and lacked NF-κB signaling dependent upon the CARMA1, BCL-10 and MALT1 signalosome. Transduction with wild type *MALT1* cDNA corrected the observed defects.

**Conclusions:**

Our nonconsanguineous patient with early onset profound combined immunodeficiency and immune dysregulation due to compound heterozygous *MALT1* mutations extends the clinical and immunologic phenotype reported in 2 prior families. Clinical cure was achieved with mixed chimerism after nonmyeloablative conditioning and HCT.

**Electronic supplementary material:**

The online version of this article (doi:10.1007/s10875-014-0125-1) contains supplementary material, which is available to authorized users.

## Introduction

Identification of the genetic causes of human immunodeficiencies has revealed the roles of many factors critical for human lymphocyte development and function. Combined immunodeficiencies (CIDs) listed by the IUIS Expert Committee on Primary Immunodeficiencies [1], include a wide spectrum of gene defects underlying susceptibility to bacterial, viral and fungal infections. The most profound of these, collectively termed severe combined immunodeficiency (SCID), are disorders with few to absent autologous T cells and absent cellular and humoral immune function [1–4]. In contrast, many CID gene defects do not abrogate development or release into the periphery of T and B cells, but instead disrupt pathways critical for their effector and regulatory roles; examples are ORAI-I, STIM-1 [5], and MHC class II deficiency [6]. While over 14 different SCID genes are known [7], many patients with CID without T cell lymphopenia have as yet unidentified genetic defects. Whole exome sequencing (WES) may identify molecular causes of CID.

Studies in knockout mice and human malignancies and immunodeficiencies have delineated the intracellular signaling pathways activated by engagement of lymphocyte antigen receptors and G-protein coupled receptors [8]. NF-κB, a central mediator of activation signals, translocates from the cytoplasm into the nucleus to initiate transcription of genes that bring about lymphocyte maturation, activation and proliferation [9]. NF-κB activation and signaling is in turn controlled by multiple mechanisms, one of which is the signalosome formed from assembly of CARMA1 (also called CARD11), BCL-10 and MALT1 into the “CBM” signaling complex [10, 11]. While the precise molecular mechanisms are still not completely clear, stimulation through the T cell and B cell receptors causes phosphorylation of CARMA1, recruitment of MALT1 and BCL-10, and oligomerization of components of the CBM complex [12–14]. This in turn activates the IκB kinase complex through TNF receptor-associated factor 6 (TRAF6)-mediated ubiquitination of NF-κB essential modulator (NEMO) [15–17], leading to phosphorylation and proteasomal degradation of the inhibitor IκBα and release of NF-κB. Thus, it is not surprising that defects in NEMO, CARMA1 and MALT1 have been found to cause human CID [18–23].

We describe a new patient in whom CID and immune dysregulation due to *MALT1* compound heterozygous mutations was successfully treated by allogeneic hematopoietic cell transplantation (HCT). This case in a non-consanguineous family, combined with 2 prior reports [22–24], broadens the spectrum of MALT1 deficiency disease and suggests an effective treatment.

## Methods

### Patient

After informed consent, as approved by the University of California San Francisco Committee on Human Research, the patient, his parents and 2 healthy siblings were studied with whole exome sequencing and immunological assessments.

### DNA Studies

Genomic DNA from the patient, obtained prior to HCT, and from his parents and siblings was subjected to WES. Analysis tools were similar to [25], with modifications detailed in the Supplementary methods. DNA variants were confirmed by Sanger sequencing. With parental consent, residual dried blood spots obtained in the newborn nursery were recovered from the California Department of Public Health Newborn Screening Program, and T cell receptor excision circles (TRECs) were analyzed as described [26].

### Cell Separations and Reagents

After HCT from an unrelated donor differing at a single HLA-C locus, the patient developed mixed chimerism of the hematopoietic system. Patient alleles were HLA-C *08:01, *03:04; donor alleles were *08:01, *07:02. Staining cells with monoclonal human IgM antibody (clone ID: TRA2G9) recognizing antigens encoded by C*01/*03/*04:01/*14:02, but not C*07/*08 [27–29], followed by PE-anti-human IgM (clone MHM-88), permitted separation of autologous patient lymphocytes from those of the donor by flow cytometry. For specific antibodies see Supplementary methods.

### PCR and Western Blotting

RNA was isolated from sorted autologous patient PBMCs obtained post-HCT (RNeasy kit, Qiagen), and expression of *MALT1* transcripts (primers in Suppl Table 1) was detected by PCR (Superscript III system, Life Technologies) followed by Sanger sequencing. The sorted cells were also lysed with 1 % NP-40 and analyzed by Western blotting using antibodies recognizing MALT1 (EP603Y, Abcam) and BCL-10 (H-197, Santa Cruz Biotechnologies).

### Intracellular Signaling Assays

For phosphorylation assays PBMCs or Epstein-Barr virus (EBV) transformed B cells were stimulated with 400 nM PMA and 250 ng/ml ionomycin at 37 °C, for 10 min. For cytokine assays PBMCs were stimulated for 6 h with PMA plus ionomycin; 200 ng/ml superantigen staphylococcal enterotoxin E (SEE, Toxin Technology, Inc.) plus 4 ug/ml anti-CD28 clone 9.3; or 1:500 anti-CD3 clone Leu-4 ascites plus 4 ug/ml anti-CD28. The cells were then fixed, permeabilized (Invitrogen Solutions A and B, Life Technologies) and incubated with either phospho-specific unconjugated antibodies followed by anti-rabbit-PE, or anti-mouse-FITC labeled secondary antibodies, or antibodies against IL-2 or IFN-γ. Fluorescent antibodies to relevant surface markers were included.

### Lentivirus Transduction

*MALT1* cDNA (Genecopia) was ligated into lentiviral vector MP-283: pSicoR-BstXI-EF1a-puro-T2A-mCherry, (kindly provided by Michael McManus, Lentiviral RNAi Core, UCSF). MP-283-MALT1 lentiviral supernatant prepared by transient transfection of 293 cells in DMEM medium with 10 % fetal calf serum (FCS) was used to transduce EBV cells in plates pre-coated with Rectronectin (Takara, Japan). After two 24 h infections, the EBV cells were washed and expanded in RPMI 1640 medium with GlutaMax (Invitrogen), 20 % FCS, penicillin, and streptomicin. MP-283 lentivirus prepared as above without the insert was used in parallel. Transduced cells were detected by mCherry fluorescence.

## Results

### Patient History

**Fig. 1:**
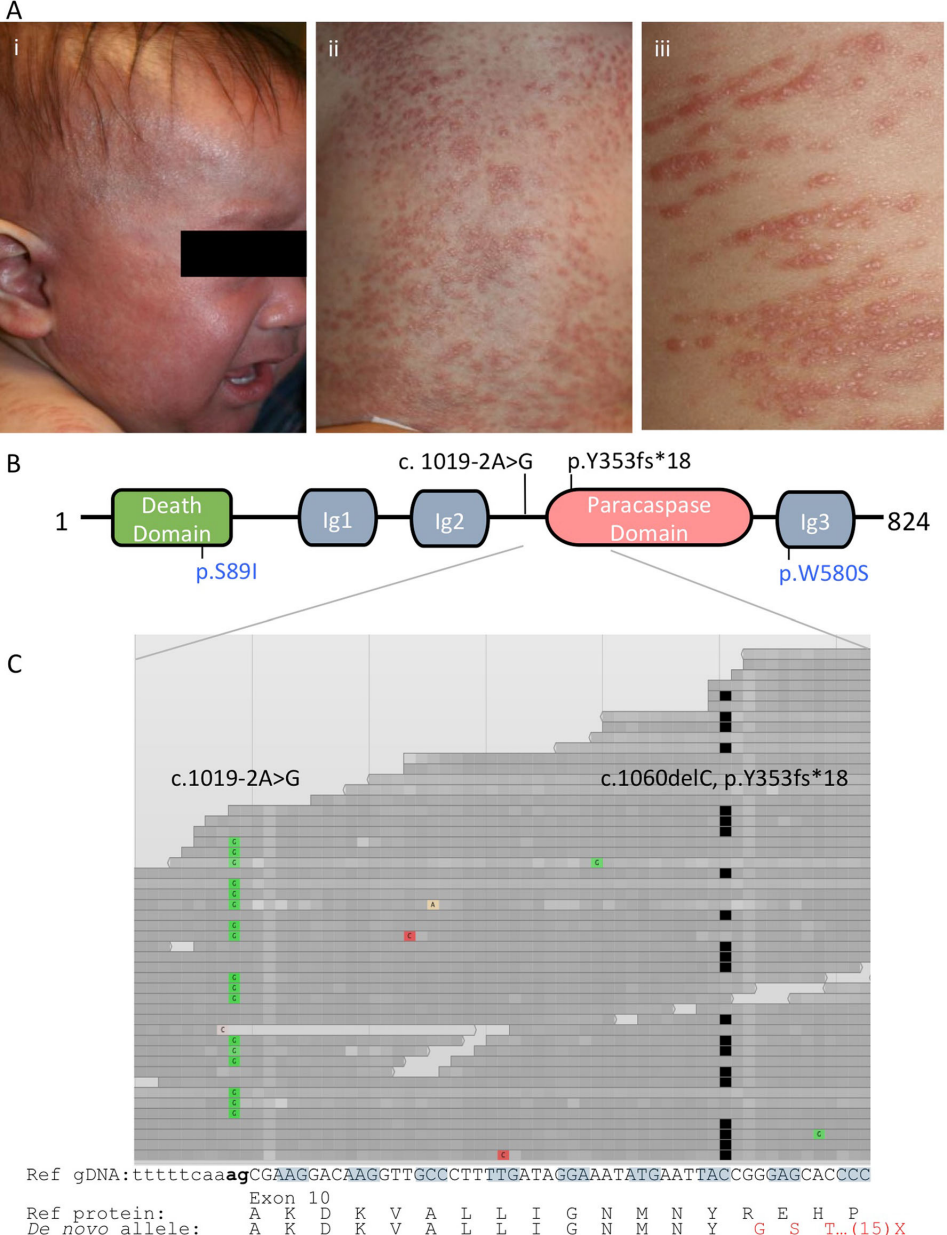
**a**, Skin lesions, showing (i) facial erythroderma (ii) dorsal trunk and (iii) close-up with linear distribution of lesions corresponding to excoriations. **b**, Schematic representation of MALT1 protein (isoform a, NP_006776.1), illustrating death domain, 3 immunoglobulin (Ig)-like domains, and paracaspase domain [30]. Mutations are shown for our patient (*black*) and prior homozygous cases (*blue*) [22, 23]. **c**, Browser view of patient stacked DNA sequence tracks, demonstrating >30X coverage with individual reads homozygous for either the splice disrupting variant c.1019-2A > G or the single nucleotide deletion cDNA c.1060delC (the former also present in maternal DNA, not shown) (ClinVar number SCV000196510). *Colored blocks*, non-reference nucleotides; *black blocks*, deleted nucleotides. Below, genomic sequence of *MALT1* exon 10 (isoform a, NM_006785.3) and protein translation with reference sequence (*black*) and *de novo* deleted sequence (*red*)

The infant, born at term to non-consanguineous parents with negative family history, developed blood-streaked diarrhea and a desquamating, erythematous pruritic rash, the latter evolving into firm erythematous papules affecting trunk, palms and soles (Fig. 1a, Table 1). There were no indications of environmental atopy; IgE was undetectable, and steroids were ineffective. Skin biopsies showed perivascular lymphocytic infiltration in the dermis. Following monthly otitis media infections, an immune workup showed expansion of CD4 and CD8 T cell populations with high proportions of naïve CD45RA T cells, but impaired in vitro proliferative responses (Tables 1 and 2). Maternal T cell engraftment was absent, and the newborn dried blood spot, retrieved from the time of the patient’s birth, had 627 T cell receptor excision circles (TRECs)/μL (normal >40). B and NK cells were present, but low immunoglobulin levels and absent antibody responses to vaccines necessitated immunoglobulin infusions. The patient experienced poor weight growth, stomatitis, oral thrush, RSV bronchiolitis, and CMV viremia and CMV pneumonitis.

**Table 1:** Clinical course, indicating infections, autoimmune manifestations, treatments (in italics) and times at which samples were obtained for study

Age	Clinical manifestation
1–3 months	Bloody stool, erythroderma (later biopsy showing lymphocyte infiltration)
9 months	Poor growth (<5 % weight, 5 % height), hospitalization for prolonged fever, presumed bacterial infections responding to systemic antibiotics; S. aureus superinfection of rash
10 months	IgG infusions instituted
13 months	Thrush, candida esophagitis
	Continuous antibiotic prophylaxis started
	DNA isolated from PBMCs, later used for whole exome sequencing
15 months	Persistent CMV >3,000 copies by PCR from blood, lung washings despite gancyclovir and foscarnet treatment; ground glass pneumonitis; self limited RSV bronchiolitis; diarrhea with C. difficile
18 months	Hematopoietic cell transplant from 9/10 HLA matched unrelated donor
19 months	Rash resolved, donor T cells detected; no graft vs. host disease
23 months	Graft vs. host disease prophylaxis discontinued
	Lymphocyte proliferation to PHA >50 % normal, persistent CMV viremia 1,500 copies
	PBMCs isolated, separated into autologous patient and donor populations for in vitro functional studies
28 months	Antibiotic prophylaxis discontinued
30 months	Donor T cell infusion for persistent CMV viremia
	CMV viremia resolved, gaining weight (25 % for age)
6 years	Donor B cell function detected with normal IgM and IgA, positive IgM isohemagglutinin

**Table 2:** Clinical and laboratory findings of patients with MALT1 deficiency

	New patient, this report		Jabara H, et al.	McKinnon M, et al.
	Pre-transplant	Post-transplant	2 siblings	1 patient
Age at immune evaluation	9 − 13 months	2.5 years	4 years, 2.25 years	15 years
Consanguinity	No		Yes	Yes
Infections				
*S. aureus*	Cellulitis	Resolved		Cellulitis, pneumonia
CMV	Blood, bronchial lavage	Resolved	Repeated urine isolations	Pneumonia
Candida	Oral thrush	Resolved	Lung, duodenum	
*C. difficile*	Diarrhea	Resolved		
RSV	Bronchiolitis	Resolved		
*S. pneumoniae*	No		Pneumonia and meningitis	Pneumonia
Other pulmonary isolates	No		*Pseudomonas, H. influenzae, K. pneumoniae*	
Other skin isolates	No		Not reported	Varicella zoster, HSV 1
Clinical manifestations				
Poor or delayed growth	Yes	Resolved (10 % height, 30 % weight for age)	Yes	Yes
Oral lesions (aphthous ulcers, cheilitis, gingivitis, thrush)	Yes	Resolved	Yes	Yes
Eczematous rash	Yes, erythroderma	Resolved	Not reported	Yes, severe dermatitis
Inflammatory bowel disease	Yes	Resolved	Yes	Yes
Neurologic development	Normal	Normal	Normal	Normal
Bronchiectasis	No	No	Yes, respiratory failure	Yes
Other findings	None	None	Mastoiditis	Dysmorphic facies, bone fractures, granulation tissue on vocal cord, larynx, ear canal
Treatments				
Immunoglobulin infusions	Yes	Yes	Yes	Yes
Antibiotics	Yes	No	Yes	Yes
Additional measures	Hematopoietic cell transplant		None reported	Nissan fundoplication, jejunostomy
Outcome		Alive, well, 7 years	Deceased, respiratory failure, 13 years, 7 years	Alive
Immunologic Parameters				
Cells X 10^9^/L (normal range for age)				
WBC (4.5−17.5)	17.0	8.9	N.A.*	N.A.
Lymphocytes (2–8)	10.6 ↑**	4.5	Normal	Normal
Eosinophils (0–1.1)	2.43 ↑	0.2	N.A.	N.A.
Lymphocyte subsets, cells/μL				
CD3 (1,610–4,230)	9,133 ↑	3,743	Normal	Elevated
CD4 (900–2,860)	4,142 ↑	2,300	Normal	Elevated
CD8 (630–1,900)	4,460 ↑	1,128	Normal	N.A.
CD4:CD8 ratio (1–2.1)	0.9 ↓	2.0	N.A.	3 ↑
CD3 CD4 CD45RA	2,526	1,426	Normal	N.A.
CD3 CD8 CD45RA	1,561 ↑	823	N.A.	N.A.
CD19 (700–1,300)	1062	226	Normal	50 ↓
NK (130–1,300)	425	451	Low, normal	Normal
T regulatory cell %				
CD25% of CD4 cells (11–20)	5 ↓	30	N.A.	N.A.
FoxP3% of CD4 CD25^hi^CTLA-4 (79–91)	56 ↓	N.A.	N.A.	Normal
CD4 CD25 CD45RA (4–67)	N.A.	7	N.A.	N.A.
CD4 CD25 CD45RO (4–25)	N.A.	23	N.A.	N.A.
B cell subset % (normal range)				
CD27 + IgM + IgD+ of CD19+ (0.2–12)	N.A.	2.1	N.A.	Absent
CD27 + IgM-IgD- of CD19+ (1.9–30.4)	N.A.	1.9	N.A.	Reduced
CD38 + IgM+ of CD19+ (7.6–48.6)	N.A.	70.6 ↑	N.A.	N.A.
CD38 + IgM- of CD19+ (2.9–51.8)	N.A.	7	N.A.	N.A.
Lymphocyte proliferation				
PHA % of lower limit of CD45 response	N.A.	57 %	Reduced	Absent
PHA % of lower limit of normal CD3 response (>50 %)	46 % ↓	53 %		
PWM % of lower limit of CD45 response (>50 %)	52 %	100 %	Reduced	N.A.
ConA % of lower limit of CD3 response (>50 %)	39 % ↓	N.A.	Reduced	N.A.
Serum immunoglobulin concentrations				
IgA mg/dL (7–13 m: 16–100; >6 y: 70–312)	15	378	Normal	Normal
IgM mg/dL (7–13 m: 25–115; >6 y: 56–352)	2 ↓	18	Normal	Normal
IgG mg/dL (7–13 m: 300–1500; >6 y: 639–1344)	160 ↓	1520 (on IgG)	Normal	Normal
IgE IU/L (<100)	<2	<1	Normal	9,856 ↑
Specific antibody titers				
Isohemagglutinins	1:2	1:4	Negative	Positive
Pneumococcal panel, 14 serotypes	0 of 14 protective		0 of 4 protective	N.A.
Tetanus toxoid	Negative		Negative	Positive
*Haemophilus influenzae B*	Negative		N.A.	N.A.
Diphtheria	Negative		N.A.	Positive
Post-transplant donor cell chimerism				
Unseparated peripheral blood		11.2 %		
CD3 T cells		16.6 %		
CD19 B cells		5.4 %		
CD14/CD15 myeloid cells		2.2 %		

*N.A., data not available

**Bold type with arrow, abnormal value

At 18 months of age, following non-ablative conditioning with 120 mg/kg cyclophosphamide, 140 mg/m^2^ melphalan and 8 mg/kg rabbit anti-thymocyte globulin, the patient received peripheral mobilized CD34-selected cells from a 9 of 10 HLA antigen-matched unrelated donor (differing at one HLA-C locus). The patient received 4.85 × 10^6^ CD34 cells/kg and 14.72 × 10^6^ CD3 cells/kg. Graft versus host disease (GVHD) prophylaxis included methotrexate and cyclosporine, and no GVHD was observed. The patient’s rash resolved within 4 weeks and did not return. Donor T cell function was established by 5 months post-HCT. However, diarrhea and CMV viremia, refractory to gancyclovir and foscarnet treatment, continued until both resolved promptly following infusion 12 months post-HCT of donor T cells that had been pulsed in vitro with CMV peptide and expanded. Immunoglobulin replacement has been given post-HCT, but donor B cell chimerism, IgA, IgM and anti-A IgM isohemagglutinins are now detectable.

### Identification of Mutations in *MALT1*

Sequencing of a comprehensive panel of known SCID genes revealed no mutations. To search for disease-causing variants, WES was performed on genomic DNA from the patient (pre-HCT) and nuclear family, with analysis under the hypothesis of an autosomal or X-linked recessive single gene disorder. The patient was a compound heterozygote with 2 non-synonymous variants affecting *MALT1* exon 10 (isoform a, NM_006785.3) (Fig. 1b and c) (ClinVar number SCV000196510) [30]. As shown, the variants were sufficiently close to one another to determine that they resided on opposite haplotypes; all sequence reads showed one or the other, but not both variants: a splice acceptor defect, cDNA c.1019-2A > G (also in maternal DNA); and a single nucleotide deletion, cDNA c.1060delC, leading to a frameshift within the MALT1 paracaspase domain and truncation after 18 missense codons, p.Y353fs*18 (not in either parent or siblings, thus *de novo* in the patient).

### Patient Mixed Donor Chimerism

**Fig. 2:**
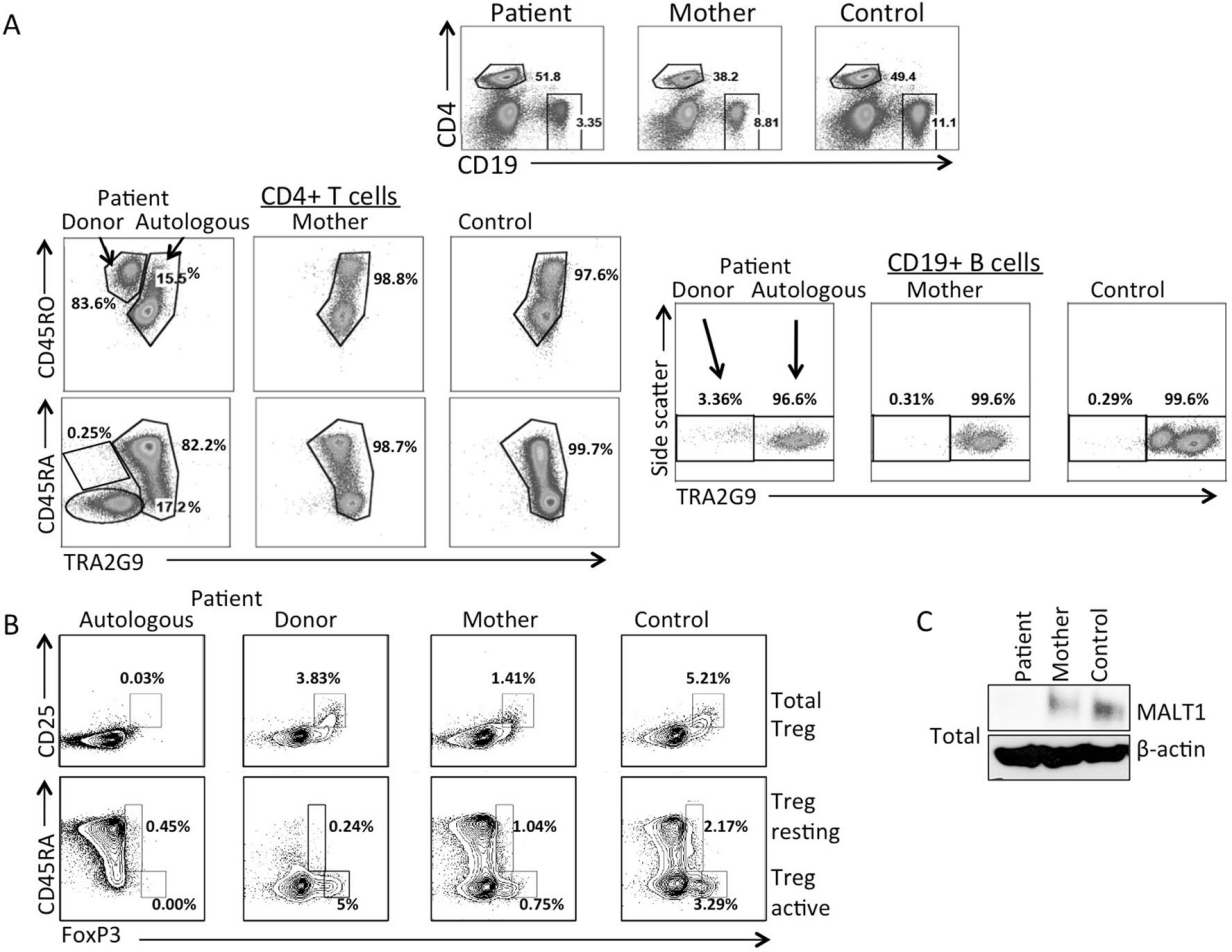
**a**, Flow cytometry of PBMCs from the patient, mother and a healthy control, using the TRA2G9 antibody to separate patient autologous cells expressing HLA-C*01/*03/*04:01/*14:02 from donor-derived cells expressing *08:01, *07:02. *Left panels*, naïve CD4+, CD45RA or CD4+ CD45RO+ T cells; *right panels*, CD19 B cells. **b**, *Upper panels*, total Treg cells (CD25+, FoxP3+); *lower panels*, resting (CD45RA+, FoxP3+) and active (CD45RA-, FoxP3+) Treg cells in PBMCs from patient autologous and donor populations, the mother; and a healthy control. **c**, MALT1 protein expression in total cell lysates isolated from PBMCs from the patient autologous cells, maternal cells and cells from a healthy control. Beta-actin was used as a loading control. All data representative of 3 independent experiments

Following HCT, mixed chimerism in the blood was established with 11.2 % of peripheral blood nucleated cells and 16.6 % of T cells being donor derived (Table 2). Using the TRA2G9 monoclonal antibody to label the patient’s, but not the donor’s, HLA-C antigens, the autologous PBMCs were distinguished by flow cytometry (Fig. 2a), showing 15.5 % donor T cells and 3.4 % donor B cells, in agreement with clinical determinations. Only 0.25 % of the donor CD4 T cells were naïve CD45RA positive cells. Pre-HCT patient CD4+ CD25+ regulatory T cells (Tregs) and FOXP3 expressing T cells were low, but were restored after HCT (Table 2). Almost all Tregs and all active Tregs in the patient’s PBMCs were donor-derived (Fig. 2b).

### Expression of *MALT1* mRNA and MALT1 Protein

Autologous, TRA2G9 antibody-positive patient lymphocyte subsets were sorted from post-HCT patient PBMCs; *MALT1* cDNA was prepared from these cells and from blood samples from the mother and a healthy control. The wild type *MALT1* exon 10 contains an even multiple of 3 nucleotides encoding a predicted nuclear export signal and allowing for the possibility of in-frame exon skipping. Indeed, short cDNA amplicons with exon 9 joined to exon 11 were detected from patient and mother, but not controls, using the internal primers listed in Supplementary Table 1. However, larger amplicons lacking exon 10 were never observed, despite robust amplification of wild type cDNA including exon 10 from controls and even from the patient in trace amounts (not shown). No cDNA from the patient c.1060delC allele could be amplified, consistent with nonsense-mediated decay of mRNA from this early truncation allele.

Whole cell lysates from autologous, sorted patient PBMCs had no detectable MALT1 protein, while maternal heterozygous cellular MALT1 levels were reduced compared to the control (Fig. 2c), further indicating that c.1060delC is a null allele.

### Functional Consequences of *MALT1* Mutations in PBMCs

**Fig. 3:**
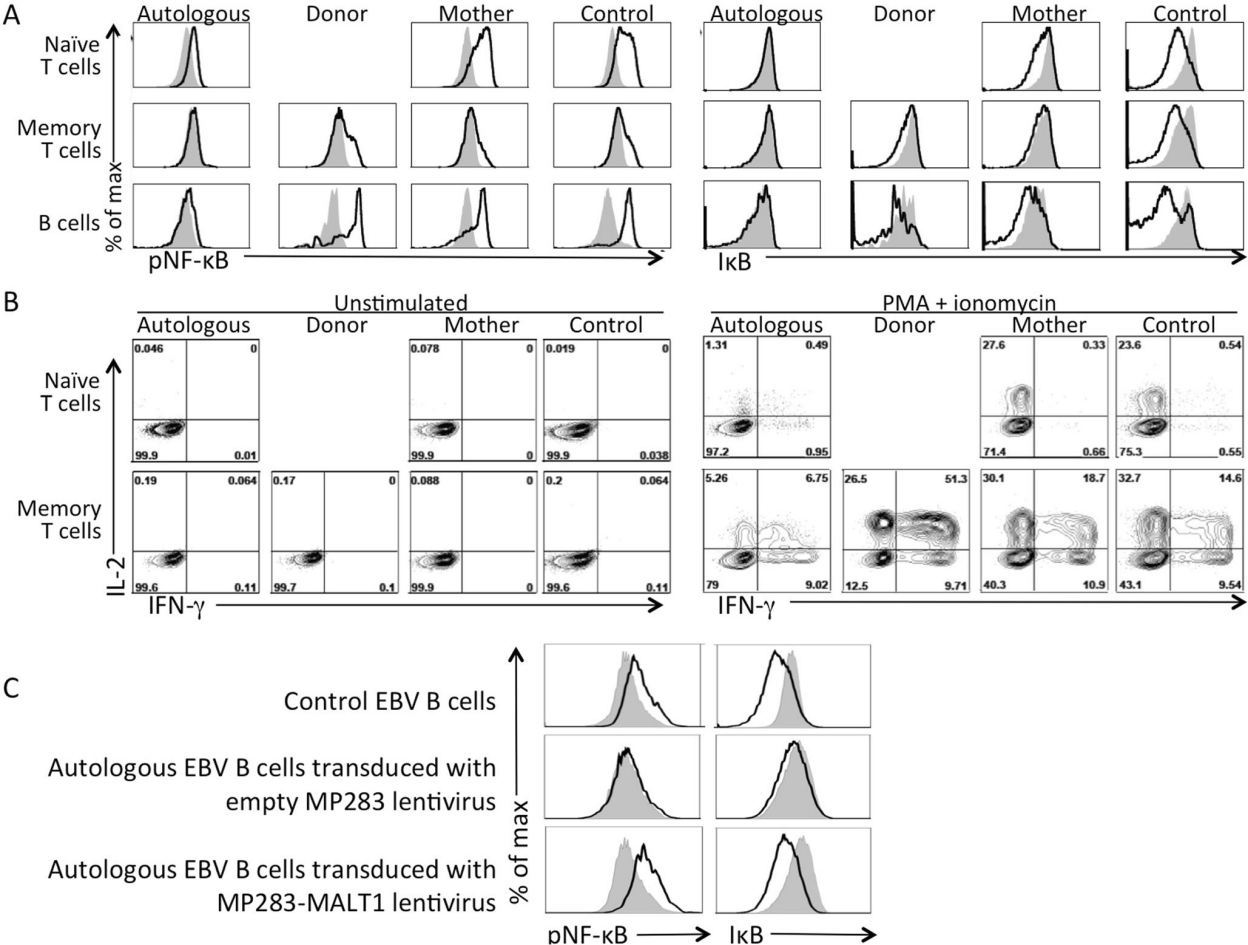
**a**, Intracellular phospho-NF-κB and IκB in gated unstimulated (*gray shading*) vs. PMA and ionomycin stimulated (*black line*) naïve and memory CD3+ T cells and CD19+ B cells from the patient, including patient autologous (TRA2G9+) and donor-derived (TRA2G9-) cells; also analyzed were cells from the mother and a healthy control. **b**, T cell expression of intracellular IL-2 (y-axis) and IFN-γ (x-axis) without (*left panels*) and with (*right panels*) PMA plus ionomycin stimulation. **c**, Analysis of NF-κB phosphorylation and IκB degradation without (*gray shading*) or with (*black lines*) stimulation with PMA and ionomycin. *Upper panels*, control EBV B cells; *middle panels*, patient autologous EBV B cells transduced with empty MP283 lentivirus; bottom panels, patient autologous EBV B cells transduced with MP283-MALT1 lentivirus. All data representative of 3 independent experiments

Phosphorylation of NF-κB and degradation of IκB following stimulation were analyzed to indicate the status of the CBM signalosome in PBMCs from the patient, his mother and a control. The patient’s autologous cells, identified by staining with the TRA2G9 antibody, were unable to phosphorylate NF-κB or degrade IκB (Fig. 3a, left and right panels, respectively), while cells from the mother and control cells demonstrated equivalent levels of NF-κB phosphorylation and IκB degradation in naïve and memory T cells as well as B cells. As noted above, too few donor-derived naïve T cells were detected in the patient’s blood to analyze. Phosphorylation of Erk, P38 and S6, each independent of the CBM signalosome, was intact in autologous patient cells, demonstrating selectivity of the MALT1 defect (Suppl Figure 1).

We also examined downstream effects of MALT1-dependent NF-κB signaling. After activation with PMA and ionomycin, donor-derived memory T cells and naïve and memory T cells from the mother and control had abundant IL-2 and IFN-γ, but the patient’s autologous, MALT1-defective T cells had little of either cytokine (Fig. 3b). Similar results were observed in PBMCs stimulated by the superantigen SEE or anti-CD3 plus anti-CD28 (Suppl Figure 2).

### Reconstitution of Impaired NF-κB Signaling in MALT1 Defective Epstein-Barr Virus Transformed B Cell Lines

A pure TRA2G9-positive MALT1 defective sub-line was expanded from sorted patient EBV cells. This and a control EBV line were analyzed at rest and following stimulation, showing the same inability as T cells to phosphorylate NF-κB or degrade IκB in response to PMA and ionomycin (Fig. 3c and not shown). To conclusively implicate *MALT1* in the functional deficits observed in CBM complex formation and downstream signaling, patient EBV cells were transduced with MP283 lentiviruses expressing mCherry alone or WT *MALT1* cDNA and mCherry. While the former had no effect (Fig. 3c, middle panels), the vector restoring *MALT1* normalized both NF-κB phosphorylation and IκB destruction (Fig. 3c, bottom panels).

## Discussion

Our patient with profound CID and dysregulation adds to the 2 prior reports and extends our understanding of MALT1-associated disease and its therapy [22, 23]. Like the prior cases (Table 2), our patient had functionally impaired T and B cells leading to recurrent bacterial and viral infections from early life, notably with CMV, which in our patient was not controlled by antiviral therapy and required post-HCT donor T cell infusions for resolution. While HCT was recently postulated as treatment for MALT1-deficient CID [24], our report is the first of a MALT1-deficient patient cured by HCT. Two prior siblings died in childhood and a surviving teenager suffers significant multi-organ disease, including T cell inflammation of the skin and bowel similar to that in our patient.

*MALT1* mutant patients reported to date had variable B cell numbers, serum immunoglobulin levels and ability to make specific antibodies (Table 2). Immune dysregulation consisting of prominent rash and suspected inflammatory bowel disease was shared between our patient and the living girl with W580S mutation [23]. In contrast to that patient, however, ours had normal B cell numbers, no IgE elevation and (like the deceased children with S89I mutation) absent protective antibody production.

Our patient’s *MALT1* compound heterozygous mutations resulted in undetectable protein and MALT1 function. As with the previous reports, after introduction of wild type *MALT1* cDNA, our patient’s mutant cells had MALT1 expression and NF-κB signaling reconstituted. Our case also highlights how *MALT1* mutations may lead to immune dysregulation and autoimmunity. After HCT, almost all Tregs and all active Tregs in our patient’s PBMCs were donor-derived (Fig. 2b), accounting for the pre-HCT failure of autologous T cells to control auto-reactive attack on the skin and possibly the intestinal tract. The mixed chimerism exhibited by our patient post-HCT allowed us to evaluate both wild type and MALT1 deficient lymphocytes that had developed from hematopoietic progenitors in vivo. Moreover, our patient’s disease resolved even though he received a non-myeloablative preparative regimen (due to his high risk status with ongoing CMV infection) that did not result in a substantial circulating naïve T cell population. Whether the donor T cells originated from limited thymic development from CD34 progenitors vs. expanded donor T cells, or a combination of both, cannot be determined. However, the patient’s successful outcome indicates that only 15 % of donor T cells were sufficient to reconstitute functional immunity and immune regulation.

The two heterozygous mutations in our patient abrogated MALT1 protein expression. Whereas the p.Y353fs*18 transcript was, as expected, degraded by nonsense-mediated mRNA decay, full-length transcripts skipping exon 10, due to the splice acceptor site defect, were also not detectable. MALT1 protein normally shuttles between the cytoplasm and nucleus with the aid of a nuclear export signal (NES1) [31, 32] and a regulatory region for NES1 encoded in exon 10, as well as second NES (NES2) in the C-terminal region of the protein [33, 34]. If a transcript missing exon 10 had been stable in our patient, the resulting protein might have been trapped in the nucleus. However, no nuclear or cytoplasmic protein from autologous patient cells was detected.

The high degree of consanguinity in the previously reported patients may indicate that loci other than *MALT1* modified their immune phenotype as well as contributing to delayed bone age, fractures, short stature, and dysmorphia in the surviving patient and poor growth in the deceased siblings; these features were absent in our outbred patient who had compound heterozygosity at the *MALT1* locus and has regained normal weight and stature following HCT.

*Malt1−/−* mice have defects in TCR activation and cytokine production similar to those observed in humans with MALT1 deficiency [10, 35]. *Malt1−/−* mice demonstrate diverse B cell defects, as have humans, with NF-κB activity in B cells reduced in one report [36], but only marginally altered after immunoglobulin receptor engagement in another [35]. Mice deficient in CBM complex proteins have reduced thymic Treg cells [37, 38], but this has not been described in mice lacking Malt1. While the recently discovered human patients with *CARD11* mutations had Treg deficiency, similar to the mouse model [20, 21], and Tregs were either normal or not studied in the previously described patients with *MALT1* mutations, our patient’s autologous PBMCs had nearly absent Tregs and very low levels of Foxp3 expression. Thus, although Malt1 appears not to be required for development of Tregs in mice, it may be important in humans, in keeping with our patient’s resolution of dysregulated immune phenotypes following HCT that provided donor-derived Tregs.

When determining the underlying cause of immune deficiencies without lymphopenia and with normal TREC numbers, signaling molecules downstream of the antigen receptors have become candidates for analysis. As shown by our patient and others with defects in MALT1, as well as patients lacking CARMA1*,* NF-κB essential modulator, and IKK2 (IKBKB) [39], proteins important in antigen receptor and NF-κB signaling should be investigated in patients with combined immunodeficiency.

## Conclusions

MALT1 deficiency can cause infantile combined immunodeficiency and immune dysregulation without T cell lymphopenia, but with impaired lymphocyte signaling through NF-κB, failure to generate memory and regulatory T cells, and hypogammaglobulinemia. Hematopoietic cell transplantation can be curative.

## Electronic supplementary material

Below is the link to the electronic supplementary material.

ESM 1 (DOCX 122 kb)

Supplementary Table 1 Primers for *MALT1* PCR (exons numbered according to isoform a, NM_006785.3) (DOCX 30 kb)

Supplementary figure 1 Intracellular FACS analysis of phosphorylation of Erk, P38 and S6 by naïve and memory CD3+ T cells and CD19+ B cells, unstimulated and after stimulation with PMA and ionomycin. Left to right: patient autologous, patient donor-derived, maternal, and healthy control cells. Insufficient donor naïve T cells were available for analysis. Gray shading, unstimulated cells; black line, stimulated cells. Data representative of 2 independent experiments. (JPEG 190 kb)

Supplementary figure 2 Intracellular IL-2 and IFN-γ expression in memory CD3 T cells, after stimulation with anti-CD3 or with superantigen, in the presence of anti-CD28 antibody. Left to right: patient autologous, patient donor-derived, maternal, and healthy control cells. Data representative of 2 independent experiments. (JPEG 190 kb)
