# Supplementary material for: Combined Immunodeficiency Due to MALT1 Mutations, Treated by Hematopoietic Cell Transplantation
Source: J Clin Immunol. 2015 Jan 28;35(2):135–46. doi: 10.1007/s10875-014-0125-1 (PMC4352191; doi:10.1007/s10875-014-0125-1)
Supplement: Supplementary file 1 — (DOCX 122 kb) [file 10875_2014_125_MOESM1_ESM.docx]

**Supplementary methods**

Analysis of whole exome sequences (WES) was similar to that described by Mallott *et al* [25] with the following programs and databases: The reads were aligned against GRCh37 (Aug 2009 release) using BWA (v 0.6.2) [40]; resulting sorted and indexed binary files had duplicates marked by Picard toolkit (v 1.81) (<http://picard.sourceforge.net>) with local realignment performed around known indel locations, and base quality scores re-calibrated using GATK (v 2.2-16) [41, 42]. Variants were called with GATK UnifiedGenotyper using exomes from this family and 34 other exomes sequenced at the same site. Variant quality scores were re-calibrated for SNPs with GATK VQSR and indel quality was annotated with filters QD <2.0, FS >200 and ReadPosRankSum <-20.0. Variants were annotated for region, effect, frequency of occurrence, and disease association using SnpEff [43] and custom scripts. Sanger sequencing for confirmation of DNA variants identified by WES was analyzed using Sequencher 4.10.1 software (Gene Codes Corporation).

Sorting and analysis of cells marked with fluorescent antibodies was performed using an LSRII flow cytometer and FACSAria II sorter (both from Beckton-Dickenson, Inc). Intracellular signaling assays used antibodies against phospho-NF-κB (clone 93H1), IκB (clone L35A5), phospho-Erk1/2 (clone 197G2), (all from Cell Signaling Technology); phospho-S6 (2F9) and phospho-P38 (pT180/pY182) (BD Biosciences); FITC-IL-2 (MQ1-17H12) and AlexaFluor647-IFN-γ (4S.B3) (Biolegend); and anti-rabbit-PE or anti-mouse-FITC labeled secondary antibodies (Jackson ImmunoResearch laboratories). PBMC sub-populations were differentiated by flow cytometry following staining for surface markers using CD25 (Clone BC96), CD19 (H1B19), CD45RA (HI100), CD45RO (UCHL1) (all from Biolegend), and CD4 (RPA-T4) (BD Biosciences).
