# Supplementary material for: Combined Immunodeficiency Due to MALT1 Mutations, Treated by Hematopoietic Cell Transplantation
Source: J Clin Immunol. 2015 Jan 28;35(2):135–46. doi: 10.1007/s10875-014-0125-1 (PMC4352191; doi:10.1007/s10875-014-0125-1)
Supplement: Supplementary file 2 — Primers for MALT1 PCR (exons numbered according to isoform a, NM_006785.3) (DOCX 30 kb) [file 10875_2014_125_MOESM2_ESM.docx]

**Supplementary Table 1:** Primers for *MALT1* PCR (exons numbered according to isoform a, NM_006785.3)

| **PCR product** | **Primer** | **Sequence** |
| --- | --- | --- |
| Full-length cDNA | Forward (from ATG initiation codon) | ATGTCGCTGTTGGGGGACCCGCTACAGGCCT |
|  | Reverse (including the 3’ stop codon) | TCATTTTTCAGAAATTCTGAGCCTGTCAGAGAA |
| exon 9-11 cDNA | Forward (within exon 9) | AGAGCAAACAACTGACCAGC |
|  | Reverse (within exon 11) | AGTCCAGTTTCTTTTTCTTGCATCA |
