# Supplementary figures and images for: Combined Immunodeficiency Due to MALT1 Mutations, Treated by Hematopoietic Cell Transplantation
Source: J Clin Immunol. 2015 Jan 28;35(2):135–46. doi: 10.1007/s10875-014-0125-1 (PMC4352191; doi:10.1007/s10875-014-0125-1)

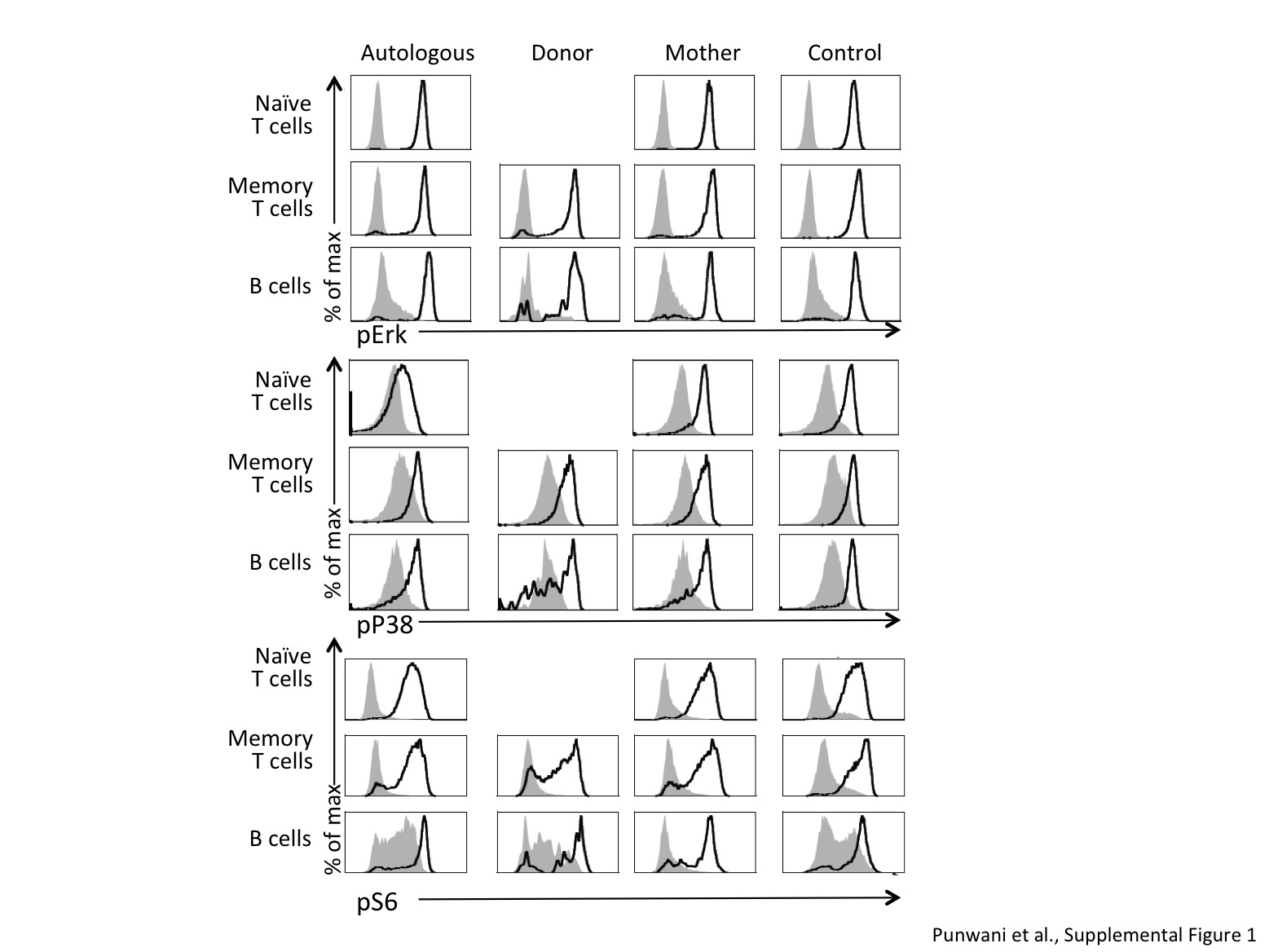

Supplement: Supplementary file 3 — Intracellular FACS analysis of phosphorylation of Erk, P38 and S6 by naïve and memory CD3+ T cells and CD19+ B cells, unstimulated and after stimulation with PMA and ionomycin. Left to right: patient autologous, patient donor-derived, maternal, and healthy control cells. Insufficient donor naïve T cells were available for analysis. Gray shading, unstimulated cells; black line, stimulated cells. Data representative of 2 independent experiments. (JPEG 190 kb) [file 10875_2014_125_Fig4_ESM.jpg]

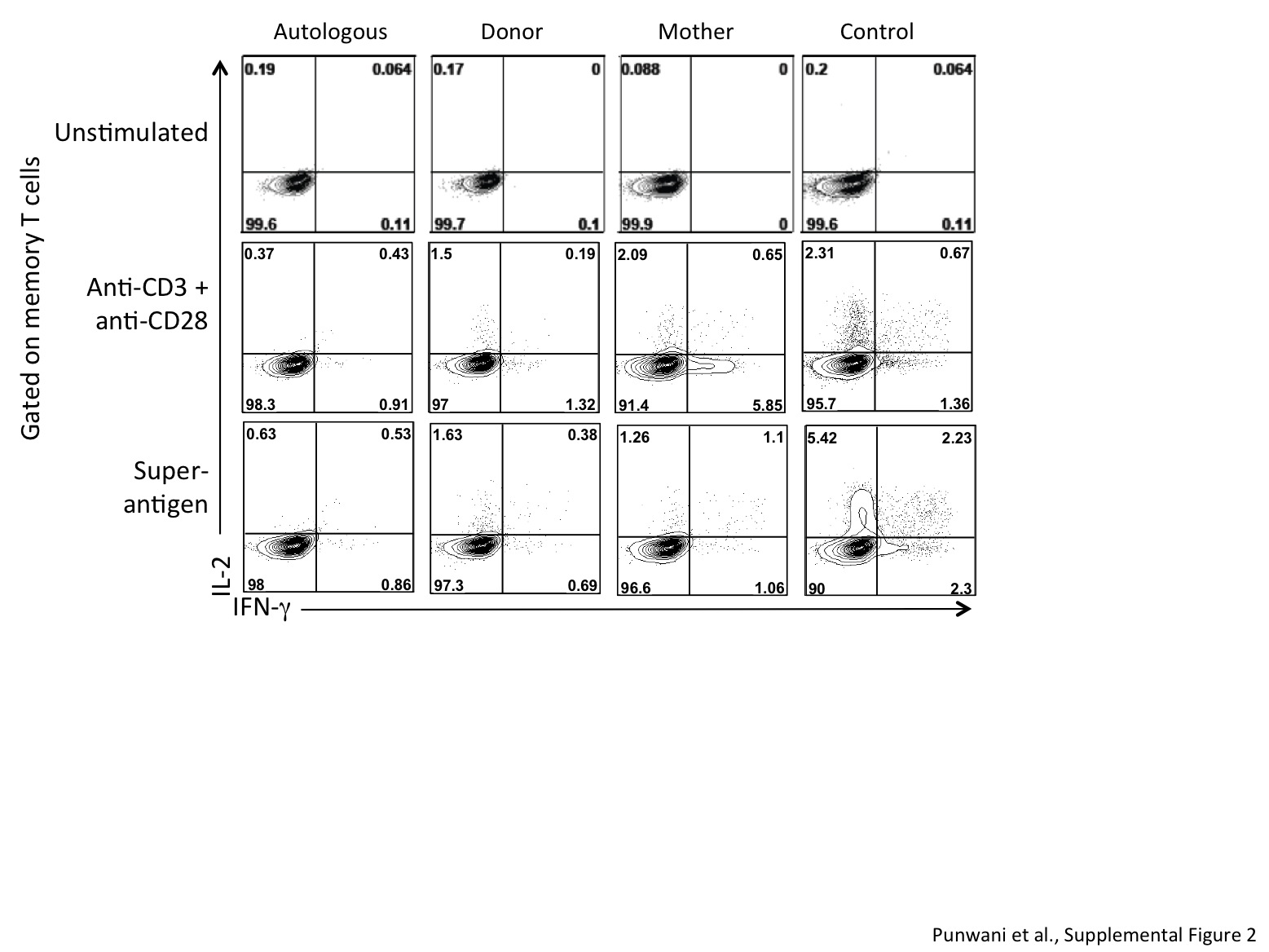

Supplement: Supplementary file 4 — Intracellular IL-2 and IFN-γ expression in memory CD3 T cells, after stimulation with anti-CD3 or with superantigen, in the presence of anti-CD28 antibody. Left to right: patient autologous, patient donor-derived, maternal, and healthy control cells. Data representative of 2 independent experiments. (JPEG 190 kb) [file 10875_2014_125_Fig5_ESM.jpg]
